# St. Bartholomew's Hospital: Precautions against Air Raids

**Published:** 1915-11-20

**Authors:** 


### THE NATIONAL GUARD AND THE HOSPITALS.

### St. Bartholomew's Hospital: Precautions Against Air Raids.

In the event of further air raids on London, the hospitals will not be found unprepared, but there is probably no institution at which more thorough precautions have been taken than at St. Bartholomew's. The scheme in operation at that hospital originated with the 1st Battalion of the City of London National Guard, whose Commandant, Colonel Cobbett, has made arrangements for a detachment of the Guard to attend at the hospital every night.

### The Guard Arrives.

At seven o'clock every evening sixty members of this unit arrive at the hospital, and at eight o'clock they are reinforced by sixty more. The first sixty remain on duty until nine o'clock, and those who come at eight stay till midnight, so that between the hours of eight and nine there are 120 men on duty, and should news be received that a raid is expected, the whole force would remain on duty until their services were no longer required. The duties of the guard are very clearly defined, and the men are prepared for them by regular drills. It may be assumed that in the event of a raid on the hospital one wing only would be hit by a bomb, in which case the maximum number of patients who would have to be removed is estimated at sixty. The work of removing them would be done by the members of the National Guard on duty, and for this purpose every man is provided with an ash stretcher pole.

### Removing the Patients.

The cases that would require removal are divided into three classes—namely, (*a*) those in bed who cannot walk or be carried; (*b*) those who can be carried on the back, or by bandy chair; (*c*) those who can walk.

The mattresses of the first group at all times have under them a special canvas sheet with a wide hem on each side through which poles can be passed, thus forming a stretcher. A supply of these sheets is provided in each ward, and it is the duty of the sister to see that one is placed under the mattress of every bed occupied by a patient who is too ill, or is otherwise unable to walk. Each of these beds is indicated by a crimson tape, so that the stretcher-bearers would know at a glance which patient they had to remove on the improvised stretchers. The second and third group would be directed and aided to leave the wards by the emergency staircases at the ends of the wings while the stretcher cases were being removed by the main staircase.

The destination of the patients on removal from a wing would depend upon circumstances; it would either be to another wing or to the surgery. In the event of the removal of patients becoming necessary, the sisters are instructed to see that all doors giving access to the wards and to the emergency staircases are opened.

To give a better idea of how precise the arrangements are it should be added that each night printed forms are filled in showing the number of patients on each floor of each of the wings. These are in the following form, but, of course, much larger:—

**Number of Patients to be Removed from Wards.**
(*a*) by stretcher; (*b*) requiring help.

FIRST FLOOR:

| | Names of Wards. On Right. | (*a*) | (*b*) | On Left. | (*a*) | (*b*) |
|---|---|---|---|---|---|---|
| East Wing ...... | Pitcairn ... | | | Henry ... | | |
| South Wing ... | President... | | | Mark ... | | |
| West Wing ... | Annie Zunz | | | Hope...... | | |

| | | (*a*) | (*b*) |
|---|---|---|---|
| Casualty Wing............... | Lucas ...... | | |

It is fervently to be hoped that such a tragedy as the dropping of a bomb on St. Bartholomew's will never occur, but the authorities have acted with great wisdom in making these admirable arrangements. Rumours that Zeppelins are about are not infrequent, and it needs no effort to imagine that such reports, notwithstanding that they are generally untrue, have a disturbing influence on those whose duties lie in the Eastern Counties. But there is certainly much comfort in the knowledge that six score trained men are keeping watch and ready to help at the first signal of danger. It also gives a sense of security to hear the tread of the sentries as they walk the quadrangle. Should an official notice be received at the hospital that an air raid is expected the sisters will be informed, so that they can disregard all unfounded rumours. Either Captain Girling Ball, the medical officer in charge of the military wing, or Mr. Hayes, the chief resident administrative officer, is always on duty in the hospital to receive any warning, so that there will be no delay in taking action.

In addition to utilising the services of the National Guard, it need hardly be said that the hospital authorities have made supplementary arrangements for dealing with outbreaks of fire. For instance, there is a hospital fire brigade under the direction of the resident fireman; and there is also a volunteer fire brigade of students. Further, the nurses are practised in the use of hand-pumps and the hospital is very adequately provided with electric fire alarms, fire hydrants, hose, and chemical extinguishers. The arrangements are that upon an outbreak of fire information is given to the porter, who communicates with the London Fire Brigade, and a horn is sounded in the square to call up the hospital fire brigade. In the case of an air raid the services of the London Fire Brigade might not be immediately available, and if the hospital were struck by incendiary bombs these arrangements might be inadequate. Hence the wise policy of the authorities in availing themselves of the services of the National Guard. The action taken at St. Bartholomew's is recommended to all hospitals in the Zeppelin zone.

